# Exploring factors constraining utilization of contraceptive services among adolescents in Southeast Nigeria: an application of the socio-ecological model

**DOI:** 10.1186/s12889-020-09276-2

**Published:** 2020-07-25

**Authors:** Uchenna Ezenwaka, Chinyere Mbachu, Nkoli Ezumah, Irene Eze, Chibuike Agu, Ifunanya Agu, Obinna Onwujekwe

**Affiliations:** 1 Health Policy Research Group, Department of Pharmacology and Therapeutics, College of Medicine, University of Nigeria Enugu-Campus, Enugu, Nigeria; 2 Department of Health Administration and Management, Faculty of Health Sciences and Technology, University of Nigeria Enugu-Campus, Enugu, Nigeria; 3 Department of Community Medicine, College of Medicine, University of Nigeria Enugu-Campus, Enugu, Nigeria; 4 Department of Community Medicine, Alex-Ekwueme Federal University Teaching Hospital, Abakaliki, Ebonyi Nigeria

**Keywords:** Adolescent, Sexual and reproductive health, Barriers, Contraceptive, Services

## Abstract

**Background:**

High rate of teenage pregnancy in Nigeria is potentially an indication of poor access to and utilization of contraceptives among this age group. This paper presents findings from in-depth exploration of perceived barriers to utilization of contraceptive services by adolescents.

**Methods:**

A qualitative study was conducted in six communities in Ebonyi state, southeast Nigeria. Eighty-one in-depth interviews and six focus group discussions were conducted. Respondents comprised policy makers, community leaders, health service providers and parents of adolescents. Pre-tested interview guides were used to collect information on perceived barriers to utilization of contraceptive services by adolescents. All interviews were audio recorded and transcribed in English. Data was analysed using thematic framework approach, and the socio-ecological model was adapted for data synthesis.

**Results:**

Individual level factors that limit access to contraceptives for adolescents include lack of awareness and poor knowledge, fear of side effects, low self-esteem, and inability to afford cost of services. Interpersonal (family-related) barriers to access include poor parent-child communication of sexual and reproductive health matters and negative attitude of parents towards to sexuality education for adolescents. Health systems barriers to accessing contraceptives for adolescents include lack of privacy and confidentiality, stock-out of contraceptive commodities, judgmental attitude of health workers, insufficient staff that are skilled in adolescent sexual and reproductive health. Gendered cultural norms, societal shaming and religious intolerance also preclude adolescents from accessing and using contraceptive services. Wider societal factors such as negative peer and media influence, absence of sexuality education in schools, lack of social networks in communities; and macro level factors such as poor economic conditions were also perceived to limit access to contraceptives for adolescents.

**Conclusion:**

Utilization of contraception is constrained by an interplay of factors acting at various levels. Addressing these barriers could contribute to improved access to contraceptive services for adolescents, as well as reduction in unwanted teenage pregnancy.

## Background

Adolescents and young people make up a considerable proportion of Nigeria’s population. It is estimated that by 2025, there will be 57 million young people in Nigeria [1]. Evidence shows that approximately 28% of Nigerian adolescents are sexually active with age of sexual debut ranging from 10 to 15 years [2]. Results from Nigeria’s Demographic and Health Survey indicate that 19% of adolescent girls aged 15–19 years have started childbearing, and 30% have had a child by the age of 19 [3]. These pregnancies are mostly unintended and have negative impact on adolescents’ health and well-being. Adolescents are more likely to die from pregnancy-related causes when compared to older women [4]. Complications of pregnancy and childbirth is the leading cause of deaths among girls aged 15–19 years in low and middle-income countries (LMICs), and it accounts for 99% of global maternal deaths [5]. Similarly, babies of adolescent mothers have higher risks of pre-term delivery, low birth weight and severe health conditions that could result in neonatal death [6]. Unwanted teenage pregnancy also has adverse social and economic consequences for adolescents. It jeopardizes future educational achievements and reduces economic viability of adolescent mothers [6, 7].

A major contributing factor to unwanted teenage pregnancy in LMICs is poor access to contraceptive services [8–10]. For instance, contraceptive prevalence rate among Nigerian adolescents is lowest when compared to other age groups [3, 11, 12]. Evidence shows that 23 million adolescent girls aged 15 to 19 years in developing countries have unmet needs for modern contraceptives, and this exposes them to higher risks of unintended pregnancy and unsafe abortions [13]. These risks and their consequences could be partly averted if their contraceptive needs are met [6].

Provision of contraceptives and other sexual and reproductive health services to adolescents are proven to produce positive health outcomes [14]. Health interventions that combine demand-creation activities and provision of contraceptive services have the potential to increase uptake among adolescents [15]. However, many adolescents in sub-Saharan African (SSA) still underuse such services where they exist [16, 17]. Considerable numbers and proportion of adolescents who are aware of and intend to use contraceptives, face numerous constraints doing so [6]. Demand and supply-side factors relating to knowledge, perception and attitude towards contraception have been attributed to limit access to sexual and reproductive health services for adolescents [6, 18].

In-depth exploration of contextual influences on access and utilization of contraceptive information and services by adolescents is vital for understanding the barriers. Exploring the perspectives of different categories of actors who play various roles in the lives of adolescents will provide insights into several inter-related factors that constrain access to contraceptive services for adolescents. This is important because these actors/factors could influence how adolescents utilize such services. Thus, addressing these barriers will promote uptake of contraceptive services for adolescents. This study therefore, examined perception of adolescent’s influencers and decision-makers on factors constraining utilization of contraceptive services among adolescents in Ebonyi State, Nigeria.

This paper provides information that will be useful to policy makers, programme managers, facility managers, development partners and advocates of adolescent health and well-being. The information would help in identifying barriers to contraceptives and means of improving the access to contraceptives for adolescents. Hence, the paper is important because without identifying these barriers so that they can be addressed, they are likely to continue to hinder effective utilization of contraceptive services among adolescents. Expanding access to contraception provides opportunities for addressing poor access to contraception among adolescents.

## Methods

### Conceptual framework

The socio-ecological model was adapted to explain factors constraining utilization of contraceptive services by adolescents [19]. The model recognizes that there are multiple interacting layers of environmental, social and communal factors that influence human behaviour. At the core of the model is the individual whose behaviour is influenced by personal knowledge, beliefs and attitudes. The second layer of the framework signifies interpersonal factors such as formal and informal social networks and social support systems including family support and relationship with health workers. The next layer shows community-level factors such as relationships between organizations, institutions and resources that embody likely sources of communication and support. The fourth layer represents organizational systems, characteristics and norms, and rules and regulations that constrain individual behaviour, while the outermost layer signifies local, state and national policies, strategies and guidelines [19].

### Study area

The study was conducted in six communities in Ebonyi state, southeast Nigeria. It has an estimated land area of 5533 km^2^. The 2017 population estimate of Ebonyi is 6,268,003 inhabitants. It has an estimated adolescent birth rate of 8.2% [3].

### Study design

This was a qualitative study that employed an exploratory approach to gain insight into adolescent’s influencers or decision-makers perception of contextual factors constraining utilization of contraceptive services among unmarried adolescents. The data was collected between October 2018 and February 2019.

### Study population and selection of participants

The study population comprised policy makers, legislator, program managers, implementing partners, local non-government organizations, community leaders, health service providers and parents/caregivers of unmarried adolescents.

Participants were purposively selected either based on their involvement in adolescent sexual and reproductive health programming in Ebonyi State, or based on their roles as key influencers of the sexual and reproductive behaviour of unmarried adolescents’. The purposive sampling sought to achieve representation of various groups (including genders) and diverse perspectives. Key influencers of adolescents’ behaviour were defined in this paper to include community leaders, school teachers and guidance counsellors, parents and caregivers, and health service providers. Other participants were recruited from; i) State government ministries and agencies including Ministry of Health, Ministry of Women Affairs and Social Development, Ministry of Youth Development, Ministry of Education and Universal Basic Education Board; ii) State House of Assembly; and iii) Non-governmental Organizations and Civil Society Organisations. Some participants were identified prior to data collection through stakeholder mapping. At that start of the project, an inception workshop was organized with key stakeholders in adolescent health in the study site. One of the objectives of the workshop was to map participants’ perspectives of key role players and influencers of adolescent sexual and reproductive health, and this information was used in determining the study population for the qualitative interviews.

A total of 81 in-depth interviews (IDIs) and 6 focus group discussion (FGDs) – comprising 8–11 participants/group were conducted. This sample represented various groups (and gender) of key influencers from each community as well as other participants in adolescent health programming. Participants’ characteristics are summarized in Table 1.

**Table 1:** Characteristics of IDI and FGD participants

IDI respondents (policymakers and influencers of adolescent SRH)		FGD participants (Village heads)	
Variables	Frequency	Variables	Frequency
**Sex**		**Sex**	
Male	42	Male	62
Female	39	Female	0
**Category of respondent**		**Occupation of respondents**	
Policymakers/Program managers	31	Traders and artisans	9
Health workers	19	Farmer	41
Parents of adolescents	8	Civil servants (including teachers) and public servants	7
Community leaders	17	Clergy	1
Religious leaders	6	Retired	4
**Level of operation**			
LGA/Community	56		
State	25		

### Data collection

Data was collected by trained qualitative researchers with skills and experience in conducting qualitative study. The choice of data collection tool (that is IDI or FGD) for the groups of participants was made based on researchers’ experience of the possibility of accessing respondents. For instance, village heads were interviewed using FGDs because experience shows that they are likely to set aside time to attend group discussions; whereas health workers were interviewed individually because due to their busy schedules, they are unlikely to leave their facilities for a long period of time to attend a group discussion. Information on barriers to utilization of contraceptive services among unmarried adolescents was collected using a pretested semi-structured IDI and FGD guides (Additional files 1 and 2) developed for the purpose of the study. The guides explored perceived barriers to accessing contraceptive information and services among unmarried adolescents. The IDI participants were interviewed at convenient locations for respondents such as health facilities, schools, offices and homes, while the FGDs were conducted either in the health facility or a central place such as the house of a villages head as convenient for the participants. Interviews were conducted in English or Igbo language, according to respondents’ preferences. Average times spent on interviews were 50 min for IDIs and 80 min for FGDs.

Prior to commencing the IDIs and FGDs, all participants were informed of the objectives of the study. Written consents were obtained from all participants having informed them of the purpose of the study, benefits and risks of participating in the study, and their rights to voluntary participation and confidentiality of data. Written consent was obtained from each participant prior to interviews/discussion. Permission to audio-record interviews/discussions was also obtained.

### Data analysis

All audio files were first transcribed in the language of the interview, and then translated to English language, if necessary. Transcripts were compared with field notes to ensure completeness and accuracy. Two independent researchers read each transcripts several times to gain full insight of the text pattern. Texts were double reviewed and discrepancies were resolved. This was done to ensure inter-coder reliability. After completing the coding, similar or related codes were grouped into categories and the categories grouped into themes, which were used in interpreting and reporting the findings. Texts were assigned to themes deduced from the five levels of social-ecological model of human behaviour namely, i) individual factors; ii) interpersonal factors; iii) community- cultural and religious factors; iv) organizational -health systems factors; and v) societal factors.

## Results

Based on the socio-ecological model that guided the study analysis, the results are presented below according to the different themes as described in the sub-section on data analysis.

### Individual level barriers of adolescents’ access to contraceptive services

Individual level factors that limit access to contraceptives for adolescents include lack of awareness and poor knowledge of contraception, fear of side effects of contraceptives, inability to afford cost of services, timidity, nonchalance and low self-esteem. Each of these factors is discussed in detail below.

#### Poor awareness and knowledge of contraception

Respondents were of the opinion that awareness of contraceptives and their sources is low among adolescents and this constitutes a major barrier to their utilization of contraceptive services. This poor awareness was linked to low level of education among adolescents in Ebonyi state.

*“Some of them (adolescents) are not aware of contraceptives and those aware don’t know where to get it from or which one will be good for them”* (IDI- state program manager, female).

*“.......because when somebody is educated they should be able to know that as a girl, I need to do this … I need to do that; even when your parents did not tell you, you must have learnt it (contraceptive methods) somewhere”* (IDI- state program manager, female).

#### Fear and experience of side effects of contraceptives

Respondent’s opined that adolescent’s fear of side effects associated with some contraceptive methods is a barrier to utilizing contraceptives. Such side effects often reported by some adolescents as a contributory factors discouraging them from using some modern contraceptive methods include prolonged or irregular menstrual cycle and weight gain/loss.

#### Inability to afford the cost of contraceptives and contraceptive service

The direct and indirect costs associated with accessing contraceptive services from formal health service providers were perceived as barriers to accessing contraceptive services. Specific mention was made of lack of funds for medical expenses and transportation fare to facilities. Although contraceptives commodities are provided free of charge in public health facilities, some remote areas have not been reached with contraceptive services and adolescents either have to pay for these commodities from private facilities or travel far distances to public facilities.

*“Adolescents may not have the fund for his/her medical expenses … … Most of the communities are far from health facilities”* (IDI- state policy maker, female).

*“Adolescents whose homes are in the remote areas find it difficult to go for SRH program. And in some of these seminars you are required to register and pay certain amount. People who don’t have money, will lose the opportunity of benefiting from such programs”* (FGD- community village heads, male, farmer).

#### Timidity, nonchalance and low self-esteem

Adolescents were reported to have low self-esteem and lack confidence to seek contraceptive services. They were also perceived to be too shy to openly discuss contraception with health workers. Some respondents perceived adolescents as having an off-hand attitude to prevention of unwanted pregnancy, hence the low demand for contraceptives.

*“They (adolescents) feel shy to open up and say the situation of things for them”* (IDI- community leader, male)

*“Adolescents have nonchalant attitude/behaviour towards getting the right information and services because even when the government organizes free seminars many of them don’t go. They feel less concerned”* (FGD- community village heads, Male, trader).

### Interpersonal barriers of adolescents’ access to contraceptives (family-related factors)

Interpersonal (family-related) barriers to contraceptive access for adolescents include poor parent-child communication of sexual and reproductive health matters and negative perceptions about adolescent sexuality education including information on contraceptives.

#### Poor parental communication of sexual and reproductive health matters with adolescents

There is a ‘culture of silence’ among parents on discussing sexual and reproductive health matters with adolescents. Some parents are not educated themselves on adolescent sexuality and do not understand why they need to discuss sexuality with their adolescents. Some other parents have negative perceptions and attitudes towards adolescents receiving sexuality education and contraceptive information and services. Hence, they tend to shy away from having these discussions.

*“Most of the time, we parents shy aware from telling our children the truth like if you sleep with a man you can get pregnant. Because we fail in this responsibility, they hear it from their peers and most of the time they get the wrong information”* (IDI- state policy maker, male).

Common reasons given for avoiding or delaying such discussions with adolescents include, i) adolescents are too young and their innocence should be protected, ii) discussions will lead to promiscuity, and iii) discussing contraception with adolescents is morally wrong.

*“Even up till now, families think that it is an abomination to talk about contraceptives among adolescents because it will make them, especially the girls … to be wayward or promiscuous; that if you start telling them how to prevent pregnancy, it means you encourage them to start having sex”* (IDI- state policy maker, male).

*“Parents will not allow you educate their children. They think that you have come to corrupt their children”* (FGD- community village heads, male, farmer).

These parents were said to be influenced by their religious and cultural believes about adolescents’ sexuality.

*“The family that claims to be too religious can be a barrier”* (IDI- state policy makers, female).

### Community *-* cultural, societal and religious barriers to adolescents’ access to and use of contraceptives

Gendered cultural norms, societal shaming and religious intolerance preclude adolescents from accessing and using contraceptive services.

#### Gendered cultural norms

Adolescent girls who seek contraceptive information and services are viewed as wayward and this hinders them from using available services in public health facilities. Gendered cultural norms were reported as fostering ignorance, naivety and timidity among adolescent girls, because it limits their access to information about contraception and sources of contraceptives.

*“Nigerian culture really affects many of our adolescents especially the girls from accessing sexual and reproductive health services greatly”* (IDI- community leader, female).

*“In our environment, a boy can go and buy condom but a girl will find it difficult because of some cultural issues”* (IDI- state policy maker, female).

#### Cultural and religious norms

Cultural norms about early contraceptive use were perceived to be linked with poor contraceptive use among adolescents. A community leader noted that use of contraceptives among adolescents is culturally unacceptable because it will prevent pregnancy and lead to reduction in population size.

*“Culturally, they look at contraceptives as a means of reducing population, and no one will like to reduce their population. That is why in our setting here, you see a man marrying so many wives to get as many children as possible”* (IDI- Traditional leader, male).

Cultural and religious beliefs constitute barriers to utilization of contraceptives among unmarried adolescents. Sexual intercourse and contraceptive use among unmarried adolescents is considered a cultural taboo. Similarly, various religions advocate for total sexual abstinence among unmarried people and view pre-marital sexual intercourse as immoral. Hence, contraception for adolescents is not discussed or tolerated. This intolerance makes adolescents who need contraceptives to go into hiding for fear of being recognized while seeking information and services.

*“There are things that culture does not permit a child to do, so if adolescents want to get access to contraceptive services and information, they will be feeling somehow (afraid). They will hide to get access because they know that our culture is against it (adolescents accessing contraceptive information and services)”* (IDI- state policy maker, female)

*“In religious settings, they look at it (contraceptive services) as a sin”* (IDI- Traditional leader, male).

*“Biblically, it is wrong to tell a girl or a boy that whenever you want to have sexual intercourse go and get condom so that you will be protected from STI or pregnancy”* (IDI- state program manager, female).

#### Societal shaming

Societal intolerance for contraceptive use among unmarried adolescents results in stigmatization and discrimination which adversely affect adolescents’ demand for contraceptives.

*“Perception of the society for the young individual is that it is totally a taboo to access contraceptive services”* (IDI- community leader, male).

#### Misconceptions about early contraceptive use

Misconceptions that contraceptive use among adolescents increases risk of infertility and limits family size were perceived to constrain adolescents from using contraceptives. Providing any form of contraceptives to unmarried adolescents was largely perceived as inappropriate because it encourages pre-marital sex and promiscuity.

*“Our culture tells us that we are not to tell a child what to do to avoid unintended pregnancy. If you are saying such things, people around will see you as abnormal [frowns]”* (IDI- state program manager, female).

### Organizational - health system barriers to adolescents’ access to and use of contraceptives

#### Unfriendly and judgmental attitude of healthcare providers

Generally, respondents perceived that unfriendly and judgmental attitudes of some healthcare providers discourages adolescents from seeking contraceptive services from health facilities. Such attitudes include: yelling, scolding, and refusal/ denial of services. Adolescent are therefore reluctant to utilize contraceptive services and uncomfortable to disclose their contraceptive needs to some health workers.

*“Some health workers will ask them their age and why they have come to ask for information about family planning when their mother is the head of the women organization. This makes them to shy away from accessing services from there* [health facilities]” (IDI- state program manager, female).

*“Let’s take for instance that an adolescent walks into the health facility and demands for a condom, everybody will shout, what are you using it for, so you are spoilt already”* (IDI- state policy maker, female).

#### Lack of privacy and confidentiality

Provider and client consultation is a crucial first step in implementing adolescent sexual and reproductive health services. This should be done with the assurance of privacy and confidentiality of information provided. However, most respondents indicated that lack of privacy between adolescents and the health providers is a key barrier to accessing and utilizing contraceptive services. When privacy and confidentiality are not ensured it leads to lack of self-confidence and trust on health workers thereby affecting utilization rate.

*“When they* [adolescents] *want to get access to these* [contraceptive] *services, they will want to trust you to keep their information confidential because they* [adolescents] *don’t like to be discussed. Once you don’t keep this they will run away from you or the facility”* (IDI- state policy maker, female).

#### Weak institutional support for youth-friendly centres

Respondents opined that weak institutional support for youth-friendly centres constrains service delivery to adolescents. This is reflected in inadequate number of youth-friendly centres, unfriendly facility settings, poor funding of available centres, lack of necessary equipment and edutainment materials (such as magazines and games) that will attract adolescents to the facility, frequent stock-out of contraceptive commodities, and insufficient number of skilled/trained health workers.

Respondents highlighted that there are very few youth-friendly centres in Ebonyi state, and services provided at these centres are limited. Hence, adolescents have to travel far distances to access contraceptive services which may not be available at the time of their visit. This was believed to discourage adolescents who require these services from utilizing them

*“There is adolescent and youth-friendly centre at Onueke* (a community) *and it is difficult for somebody at Ngbo* (another community) *to get access to service there because of the distance”* (IDI- state policy maker, male).

The design of adolescent health units in some public health facilities were thought to be unfriendly and poorly set-out to ensure privacy for adolescents. In some public facilities, it was reported that adolescent health units were proximate to adult clinics and this makes adolescents reluctant to use such facilities for fear of being recognized and stigmatized.

*“Adolescents do not like open services where adults can easily see them … they like places that are secluded”* (IDI- state program manager, female).

Inadequate number of health workers who are trained to handle adolescent sexual and reproductive health was identified as a barrier to adolescents’ accessing contraceptive services from youth-friendly centres

*“Even when health facilities are available, they do not have skilled providers who can manage adolescent sexual and reproductive health concerns”* (IDI- state program manager, female).

Respondents also highlighted that frequent stock-outs and unavailability of preferred contraceptive methods in youth-friendly centres adversely affects access to and utilization of contraceptives by adolescents.

### Societal level barriers to adolescents’ access to and use of contraceptives

Negative peer and media influence, absence of sexuality education in schools and lack of social networks in communities were highlighted as some societal level barriers to adolescents’ access to contraceptive information and services.

#### Influence of peer, mass and social media

These views of peers sometimes prevent sexually active adolescents from utilizing contraceptive services and respondents viewed this as negative peer influence. A public secondary school principal stated that, *“In the school, peers influence adolescents negatively because they may tell them that if they go for contraceptive counseling they may be seen as spoilt people”* (IDI- LGA community leader, female)

Social and mass media were also reported to have a significant influence on adolescents which could result in poor utilization of contraceptive information and services.

#### Incomprehensive sexuality education in school curriculum

It was reported that topics on sexuality education and contraception are not provided to secondary school students in their curriculum because of policy restrictions and societal inhibitions on teachers. Some respondents, perceived this denial of contraceptive information as wrong and unhelpful considering high levels of unintended adolescent pregnancy in the State.

*“Our policy has restricted us from talking much about contraceptive needs in the school system, which already is a problem”* (IDI- state program manager, male).

*“Some of the times even when the teachers want to provide sexuality education they do not teach it (contraception) saying that the parent-teacher association will frown at it”* (IDI- state policy maker, female).

#### Lack of social networks and community support

Respondents stated that there are no community structures such as ‘adolescent health support network’ to facilitate access and utilization of SRH services among adolescents. This network is believed to act as a linkage between the community and health facilities.

*“I don’t think there is space for adolescents that is why they are scared when they visit health centres because they only talk about mothers, children and adults … so they feel rejected”* (IDI- state program manager, female).

### Macro-context barriers to adolescents’ access to and use of contraceptives

#### Poverty level in the society

The poor economic condition in society was reported as a barrier to adolescents’ access to contraceptive services. A community influencer stated that *"poverty of the mind, poverty of the cash, poverty of the food affects adolescents …*. *for instance, when you tell someone to come for seminars and learn sex education, he will prefer to go to farm. Their parents will say, go to farm to produce what he will eat with his children. And when you tell somebody, come we have a program, they will say; give us money; if you are not ready to give us money, we are not coming. The person doesn’t know the importance. The person is seriously looking for money to solve their problem … "* (IDI- Traditional leader, male).

## Discussion

The study highlights socio-ecological contextual factors limiting adolescents’ access to and utilization of contraceptive services in Nigeria. Individual level barriers such as lack of boldness to seek contraceptive services when needed, poor knowledge of contraceptive methods and fear of side effects of contraceptives conform with previous studies reporting lack of knowledge of types and sources of contraceptives, and misconceptions about side effects of contraceptives [20–23]. These findings highlight the need to provide comprehensive information to adolescents on the types, modes of action and sources of contraceptives.

Parents reported feeling uncomfortable discussing sexuality and sex-related matters with their children. This discomfort stems from cultural and religious beliefs, and misconceptions about health and social effects of early onset contraception such as infertility and promiscuity. Similar perceptions have been reported in south-west Nigeria where parents stated that discussing sexuality with adolescents generates curiosity and interest in sexual intercourse [24]. This lack of parental communication of sex-related matters heightens the influence of external factors such as media and peers on adolescents. Others explanations for poor parental communication include lack of knowledge, poor communication skills and time constraint. These assertions were also been reported by a study that explored parental communication of adolescent sexuality and reproductive behaviour [25]. Evidence shows that dialogue between parents and adolescents is an effective method of communicating sexual and reproductive information and instilling values [21, 26, 27]. Parents have tremendous influence on a child’s emotional, social, and cognitive development, and should be involved in addressing their SRH needs [28, 29].

Societal norms that are rooted in culture and religion contribute to limiting access to contraceptive information and services for adolescents. Religion considers premarital sexual intercourse as sinful [30], and adolescents are culturally obliged to abstain from sexual intercourse until they are married. Hence, adolescents are expected to abstain from sexual intercourse until marriage. Society also perceives that exposure to contraceptives would encourage promiscuity and early contraceptive knowledge by adolescents suggests virtue erosion [31, 32]. These norms could result in exclusion and stigmatization of adolescents who need contraceptive services. Social exclusion and stigmatization of adolescents who engage in premarital sex is a major contributor to underutilization of reproductive health services and teenage pregnancies [33, 34]. Community dialogue and focus group discussions with parents and community members could be useful for deconstructing societal norms and misconceptions about adolescent sexuality and access to contraception.

The negative attitude of health providers towards adolescents seeking contraceptive services is a reason for low turnouts in youth-friendly centres. Our findings confirm reports that health workers in Nigeria and other LMICs are reluctant and unwilling to provide contraceptive services to adolescents because they do not approve of premarital sex [18, 30, 35]. This causes embarrassment for young people seeking sexuality and contraceptive information from formal healthcare facilities. Ill-treatment by health workers deters young people from seeking contraceptive services [36]. On the flipside, non-judgmental and kind health workers motivate adolescents and young people to utilize available sexual and reproductive health service [21]. Evidence shows that the Nigerian health system has inadequate numbers of health workers that are specially trained and skilled to offer adolescent and youth-friendly sexual and reproductive health services [37]. The few who have the skills are saddled with additional responsibilities which increase their work load and trigger unfriendly attitudes to clients. Hence, it is necessary to invest in strategies that would ensure sustained availability of skilled health workers who can provide sexual and reproductive health services to adolescents while maintaining ethical codes of privacy and confidentiality [38].

Other supply-side issues that constrain access to contraceptive services for adolescents such as frequent stock-out of contraceptive commodities, lack of edutainment materials and inadequate numbers of youth-friendly health centres, have been reported by similar studies [39–41]. Inadequate supply of contraceptive commodities to health centres disproportionately favours married and older women over unmarried young people such as adolescents. This is because contraceptives are mostly provided through family planning clinics and unmarried young people are not prioritized for ‘family planning’ services. The unavailability to youth-friendly centres, that are supposed to fill this gap in service delivery, further worsens the case for adolescents who need contraceptive services.

Media and peer relationships have been recognized as major influences that shape adolescent health and development [42, 43]. Commonly referred to as the ‘social contagion’, these factors begin to exert more impact in adolescence as parental and community influences wane. However, they are notorious for providing incorrect or incomplete information, instilling negative values, and encouraging unsafe sexual behaviours among adolescents. These highlight the invaluable role of parents and teachers, who make the earliest contacts with children, have to play is providing comprehensive information on sexuality and reproduction to adolescents. The inclusion of comprehensive sexuality education in school curriculum cannot be overemphasized because it provides adolescents with correct and appropriate information on the subject matter and teaches them to acknowledge other personalities and preferences.

Finally, poverty was reported to be a barrier to access to contraceptives for adolescents. This is a commonly reported determinant of access to health services, particularly in contexts where out-of-pocket payment is prevalent [44, 45]. Although contraceptives are provided free of charge in public health facilities in Nigeria, our study has highlighted several supply-side factors that discourage adolescents from seeking health services from public health facilities. This implies that majority of them would have to pay for contraceptives. Since majority of adolescents are unlikely to be employed, they are also unlikely to afford to pay for contraceptives and contraceptive services provided by private and informal healthcare providers. This strengthens the argument for establishing youth-friendly health centres to attend to the peculiar needs of adolescents and young people.

Interpersonal, community, organizational and societal factors that preclude adolescents from benefitting from sexual and reproductive health interventions have been underlined and these barriers influence how adolescents experience and in turn utilize available services. Participants’ reflections of the barriers to accessing contraceptives for adolescents show that majority of these barriers are outside of the control of adolescents themselves. Adults have a key role to play in addressing the factors that hinder adolescents from accessing contraceptives. Hence, the call to strategically involve key stakeholders in communities, schools and policy arena in changing the narrative and promoting adolescent-friendly SRH service provision [46].

A major limitation of this manuscript is that it does not report the perspectives of adolescents whose barriers to accessing contraceptive services was explored. However, in-depth exploration of perspectives of different categories of adults who play various roles in the lives of adolescents revealed barriers that are consistent with literature. It also provides insights into the inter-relatedness of several factors that constrain access to contraceptive services for adolescents. Future studies could investigate how these factors interact and the outcomes of their interactions on access to contraceptives for adolescents.

## Conclusion

Several interrelated barriers operating at individual, interpersonal, community, organizational and societal levels, hinder utilization of contraceptive services among adolescents in Ebonyi State. However, it is evident that the burden of unprotected sexual intercourse would be significantly reduced when contraceptive needs of adolescents are met, with the removal of identified barriers. Indeed, strategically engaging relevant actors/adolescents influencers in interventions to improve SRH adolescents including contraceptives is recommended for promoting adolescents SRH provision as most of the identified barriers are within their prerogative to address. Hence, addressing these barriers will increase contraceptive uptake which will reduce unintended pregnancy and other negative health and social consequences of unprotected sexual intercourse. This is crucial as it will contribute to meeting the Sustainable Development Goal (SDG 3) target of ensuring healthy lives and promoting well-being for all ages.

## Supplementary information

**Additional file 1.** IDI guide for policymakers and influencers of adolescent SRH.

**Additional file 2.** FGD guide for village heads.

## Data Availability

Some of the data generated or analysed during this study are included in this published article. Additional data are available from the corresponding author on reasonable request.

## Abbreviations

ASRH: Adolescent Sexual and Reproductive Health
FGD: Focus group discussion
IDI: In-depth interview
LMICs: Low and middle-income countries
SDG: Sustainable Development Goal
SRH: Sexual and Reproductive Health
