# Supplementary material for: Exploring factors constraining utilization of contraceptive services among adolescents in Southeast Nigeria: an application of the socio-ecological model
Source: BMC Public Health. 2020 Jul 25;20:1162. doi: 10.1186/s12889-020-09276-2 (PMC7382857; doi:10.1186/s12889-020-09276-2)
Supplement: Supplementary file 1 — Additional file 1. IDI guide for policymakers and influencers of adolescent SRH. [file 12889_2020_9276_MOESM1_ESM.docx]

## Factors constraining utilization of contraceptive services among adolescents in Ebonyi State southeast, Nigeria

### In-depth interview guide for policy makers and influencers

### Introduction, purpose and procedure

I am a trained data collector from Health Policy Research Group University of Nigeria Enugu Campus and we are working with Ebonyi State government to conduct a study on Adolescent Sexual and Reproductive Health.

The aim of this interview is to get your views on barriers/factors constraining access to Adolescent sexual and reproductive health - contraceptive information and services among unmarried adolescents in Ebonyi State. Adolescent sexual and reproductive health refers to all matters relating to their safe sex life and capability to have children. I am particularly interested in your experiences and/or perception access to contraceptives for unmarried adolescents aged 13 to 18 years. The information you provide in this interview will be treated as confidential and your participation will be anonymized.

With your permission, I would like to record this interview to make sure I accurately capture our discussion. This interview will last about 40-60 minutes.

### Background characteristics

Interview code

Date of interview

Designation of interviewee

Sex

Time start

Time stop

### Discussion

1. In your opinion or knowledge, what barriers do adolescents face in accessing SRH **information**?
   - What challenges do they face in accessing contraception information? *(****Prompt for:*** *individual family, society, cultural beliefs, religion, peer-influence, health services, financial access, etc factors)*
   - Do you have an experience on how the barriers you mentioned affect access to contraceptive information? *(If yes, tell the respondents to share the experience with you).*
2. In your opinion or knowledge, what barriers do adolescents face in accessing SRH **services**?
   - What challenges do they face in accessing contraception services and using contraceptives? *(****Prompt for:*** *family, society, cultural beliefs, religion, peer-influence, health services, financial access, etc.)*
   - How do societal and cultural norms influence access to contraceptive services for adolescents?
   - How do religious beliefs and organizations influence access to contraceptive services for adolescents?
   - Do you think that peers influence their decision to access contraceptive services? If yes, could you please tell me how?
   - What are health services barriers do you think that affect unmarried adolescents accessing contraceptive services?
3. Are there situations in which contraceptive use among unmarried adolescents is acceptable or not acceptable? Can you tell me about these situations?
4. As a parent/guardian, what kinds of discussions do you have with your adolescents about SRH matters particularly contraceptives?

*(****Prompts:*** *physiological and anatomical changes during puberty; boyfriend/girlfriend relationships; sexuality – orientation, exposure, use of protection, peer group influence, etc)*

- What challenges do you face in discussing contraceptives with your adolescents?
- Whose responsibility is it in the home (father or mother or other relative) to discuss SRH matters with adolescents? Why is this so?
- If you do not discuss contraceptive with them, what are your reasons?

***(Probe for*** *situations in which contraceptive use among adolescents is acceptable or not acceptable)*

- Can you tell me why some sexually-active adolescents do not use contraceptives? *(****Prompts****: lack of information, family support, society, culture, religion, peer-influence, health services, financial access, etc)*

**Probes:**

- - How do societal norms and perceptions about young people having sex influence access to contraceptives for adolescents?
  - How do religious beliefs and religious organizations influence access to contraceptives for adolescents?
