# Supplementary material for: Exploring factors constraining utilization of contraceptive services among adolescents in Southeast Nigeria: an application of the socio-ecological model
Source: BMC Public Health. 2020 Jul 25;20:1162. doi: 10.1186/s12889-020-09276-2 (PMC7382857; doi:10.1186/s12889-020-09276-2)
Supplement: Supplementary file 2 — Additional file 2. FGD guide for village heads. [file 12889_2020_9276_MOESM2_ESM.docx]

## Factors constraining utilization of contraceptive services among adolescents in Ebonyi State southeast, Nigeria

### In-depth interview guide for policy makers and influencers

### Introduction, purpose and procedure

I am a trained data collector from Health Policy Research Group University of Nigeria Enugu Campus and we are working with Ebonyi State government to conduct a study on Adolescent Sexual and Reproductive Health.

The aim of this discussion is to get your views on barriers/factors constraining access to Adolescent sexual and reproductive health - contraceptive information and services among unmarried adolescents in Ebonyi State. Adolescent sexual and reproductive health refers to all matters relating to their safe sex life and capability to have children. I am particularly interested in your experiences and/or perception access to contraceptives for unmarried adolescents aged 13 to 18 years. The information you provide in this interview will be treated as confidential and your participation will be anonymized.

With your permission, I would like to record this discussion to make sure I accurately capture our discussion. This discussion will last about 50-90 minutes.

### Background characteristics

Interview code

Date of interview

Background information of participants

Venue

Time start

Time stop

### Discussion

**Perception of contraception and contraceptive information and use among adolescents**

1. Should sexually active adolescents have access to contraceptives information and services? What are your reasons for saying so?

- Are there situations in which contraceptive contraceptives information and services among adolescents is acceptable or not acceptable? Can you tell me about these situations?

1. From your experience or opinion, can you tell me why some sexually-active unmarried adolescents do not have access or contraceptives **information**? *(****Prompts****: lack of information, family support, society, culture, religion, peer-influence, health services, financial access, etc)*

**Probes:**

- - How do societal norms and perceptions about young people having sex influence access to contraceptives information for adolescents?
  - How do religious beliefs and religious organizations influence access to contraceptives information for adolescents

1. From your experience or opinion, can you tell me why some sexually-active unmarried adolescents do not have access or use contraceptives **services**? *(****Prompts****: lack of information, family support, society, culture, religion, peer-influence, health services, financial access, etc)*

**Probes:**

- - How do societal norms and perceptions about young people having sex influence access to contraceptives services for adolescents?
  - How do religious beliefs and religious organizations influence access to contraceptives services for adolescents?
